# Pathways to a diagnosis of autism spectrum disorder in Germany: a survey of parents

**DOI:** 10.1186/s13034-019-0276-1

**Published:** 2019-03-21

**Authors:** Juliana Höfer, Falk Hoffmann, Inge Kamp-Becker, Luise Poustka, Veit Roessner, Sanna Stroth, Nicole Wolff, Christian J. Bachmann

**Affiliations:** 1 Department of Health Services Research, Carl von Ossietzky University Oldenburg, Ammerländer Heerstr. 140, 26129 Oldenburg, Germany; 2 Department of Child and Adolescent Psychiatry, Psychosomatics and Psychotherapy, Philipps University Marburg, Hans-Sachs-Str. 4, 35039 Marburg, Germany; 3 Department of Child and Adolescent Psychiatry, University Medical Center Göttingen, Von-Siebold-Str. 5, 37075 Göttingen, Germany; 4 Department of Child and Adolescent Psychiatry, Medical Faculty of the Technical University Dresden, Fetscherstr. 74, 01307 Dresden, Germany; 5 Department of Child and Adolescent Psychiatry, LVR-Klinikum Düsseldorf/Heinrich-Heine University Düsseldorf, Bergische Landstr. 2, 40629 Düsseldorf, Germany

**Keywords:** Autism spectrum disorder, Autism, Diagnosis, Diagnostic process, Pathways, Satisfaction, Germany

## Abstract

**Background:**

Early identification of autism spectrum disorders (ASD) is a prerequisite for access to early interventions. Although parents often note developmental atypicalities during the first 2 years of life, many children with ASD are not diagnosed until school age. For parents, the long period between first parental concerns and diagnosis is often frustrating and accompanied by uncertainty and worry.

**Methods:**

This study retrospectively explored the trajectories of children with a confirmed ASD diagnosis during the diagnostic process, from first parental concerns about their child’s development until the definite diagnosis. A survey concerning the diagnostic process was distributed to parents or legal guardians of children with ASD from three specialized ASD outpatient clinics in Germany.

**Results:**

The response rate was 36.9%, and the final sample consisted of carers of 207 affected children (83.6% male, mean age 12.9 years). The children had been diagnosed with childhood autism (55.6%), Asperger syndrome (24.2%), or atypical autism (20.3%). On average, parents had first concerns when their child was 23.4 months old, and an ASD diagnosis was established at a mean age of 78.5 months. Children with atypical autism or Asperger syndrome were diagnosed significantly later (83.9 and 98.1 months, respectively) than children with childhood autism (68.1 months). Children with an IQ < 85 were diagnosed much earlier than those with an IQ ≥ 85. On average, parents visited 3.4 different health professionals (SD = 2.4, range 1–20, median: 3.0) until their child received a definite ASD diagnosis. Overall, 38.5% of carers were satisfied with the diagnostic process.

**Conclusions:**

In this sample of children with ASD in Germany, the time to diagnosis was higher than in the majority of other comparable studies. These results flag the need for improved forms of service provision and delivery for suspected cases of ASD in Germany.

**Electronic supplementary material:**

The online version of this article (10.1186/s13034-019-0276-1) contains supplementary material, which is available to authorized users.

## Introduction

According to DSM-5, autism spectrum disorder (ASD) is characterized by persistent deficits in social communication and social interaction across multiple contexts accompanied by restricted, repetitive patterns of behavior, interests, or activities [1]. The prevalence of ASD has been steadily increasing in recent decades [2–4]. Its worldwide prevalence is now estimated at 1% [5, 6], with the administrative prevalence in Germany being reported as 0.38% [2].

Besides environmental factors, genetics play a key role in the etiology of ASD, leading to abnormal brain development. The current treatment standard in children with ASD are early interventions, which focus on the improvement of social functioning, language and communication skills, and are effective in improving the long-term outcome in the above-mentioned domains. Accordingly, early identification of young children with ASD, and subsequent access to early interventions for these children is essential [5, 7–11]. In most Western countries a timely diagnosis of ASD also enables affected individuals and their families to get access to ASD specific health and social services.

Although in most cases ASD can be reliably diagnosed at the age of 24 months [12], with many parents reporting initial concerns even much earlier, a significant proportion of children are not diagnosed until school age [11, 13, 14]. For parents, this diagnostic delay is often accompanied by a long, frustrating period of uncertainty and worry, which can increase parental stress and dissatisfaction [15–17]. However, the symptomatology of older children and adolescents with ASD is often heterogeneous, and psychiatric comorbidities may complicate the differentiation of individuals with ASD from those with other diagnoses [18–21]. Moreover, ASD symptoms can appear in a variety of behavioural disorders, e.g. attention-deficit/hyperactivity disorder, conduct disorder, or intellectual impairment [22]. Due to this symptom overlap, the diagnostic process of ASD can sometimes be difficult. For an accurate diagnosis of ASD, so-called “gold standard” psychometric instruments are employed, i.e. a standardized interview with the parents (Autism Diagnostic Interview-Revised, ADI-R), in combination with a semi-structured, standardized observation (Autism Diagnostic Observation Schedule, ADOS/ADOS-2) [4, 23–26]. Both special training and experience with these instruments are needed to apply them properly in order to reach a valid diagnosis [22, 27].

In Germany, so far no standardised pathways for diagnostic assessment in cases of suspected ASD exist. Parents of children with suspected ASD are free to consult any health professional of their choice, without any need for referral. In general, in Germany paediatricians and general practitioners are the first point of contact for parents of children with mental health concerns [28].

A systematic review [13] shows that for ASD the mean age at diagnosis varies considerably between studies. The average age at ASD diagnosis ranged here from 38 to 120 months and has decreased over time. Besides clinical characteristics, the review revealed several other factors like sociodemographic characteristics or parental concern associated with age at diagnosis.

Although there exists already a considerable body of literature on the process of obtaining an ASD diagnosis, besides a large multi-country European study [29] that included data from Germany, there are only two studies exploring parents’ experiences of the diagnostic process in Germany. One of these studies solely focussed on the time between age at first symptoms and age at diagnosis [30], while the other study included only children with Asperger syndrome [14]. Thus, for Germany, data related to professionals seen on the route to ASD diagnosis are not yet available.

Additionally, several studies have demonstrated an association between time to ASD diagnosis and the number of professionals consulted, respectively, and satisfaction of parents with the diagnostic process [15–17].

Against this background, the purpose of the study was to explore the experiences of parents of children and adolescents regarding the process of attaining an ASD diagnosis in Germany. Besides the time component of the diagnostic process, this survey also focused on the professionals consulted and the parental satisfaction with the diagnostic process.

## Methods

This study is part of a large clinical and research network, the ASD-Net, focusing on the key challenges in ASD diagnostics, therapy and health service research [4].

### Recruitment and participants

Between November 2015 and June 2016 three specialised child and adolescent psychiatric ASD outpatient clinics in Germany (Dresden, Mannheim and Marburg) contacted the parents/legal guardians of all children, who had a diagnosis of ASD and received services from or were diagnosed at the department. All patients were diagnosed using the ADOS/ADOS-2 and the ADI-R [4, 23, 24]. Inclusion criteria for the study were child’s age under 19 years and a confirmed diagnosis of pervasive developmental disorder according to ICD-10.

### Questionnaire and instruments

Parents of children with ASD completed a self-administered questionnaire on health-care services for ASD, based on the Client Service Receipt Inventory (CSRI) [31, 32], one of the most commonly used measures of health and social care service use. Together with a cover letter, a participant information sheet and a written informed consent form, the questionnaire has been usually mailed to parents. Only in exceptional cases, the survey documents were handed over personally. Depending on participating department, few weeks after the initial contact the parents received a reminder.

The participants had to give their consent, that their questionnaire data will be linked to data on, for instance, age, sex, clinical diagnosis (ICD 10-code), ADOS-2 comparison score and level of intellectual functioning of the child with ASD in a pseudonymised form. According to ICD-10, the level of intellectual functioning was divided into two groups: learning or intellectual disability (IQ < 85) vs. no learning or intellectual disability (IQ ≥ 85). We further summarized the ADOS-2 comparison score into the following three different groups: minimal to low (score 1–4), moderate (score 5–7), and high (score 9–10) [33].

In addition to demographic characteristics the questionnaire asked parents about selected features of the diagnostic process. The items concerning the diagnostic process were based on previous studies on this topic [15–17], and included (1) time taken to get an ASD diagnosis (age at first concerns, age at diagnosis), (2) professionals consulted because of first concerns, (3) outcome of first consultation, (4) professionals who made the final diagnosis, (5) number of professionals seen to get the ASD diagnosis and (6) satisfaction with the diagnostic process (using a 5-point Likert-scale, ranging from ‘very dissatisfied’ to ‘very satisfied’).

Children’s age, the respondents’ relationship to the child and the educational level of the parents, obtained from the questionnaire, were analysed for background information. To examine the highest parental educational level, participants were asked to provide information about mothers’ and fathers’ educational background. For analyses, the highest of the two levels was considered. The level of education was defined in accordance with the International Standard Classification of Education (ISCED) [34, 35] and has been classified into two groups: low/medium education (ISCED level 0–2) and high education (ISCED level 3). Referring to the German school system, low educational level complies with 9 years of schooling or leaving school without having acquired any school-leaving qualification. Medium educational level is equivalent to 10 years of schooling and high educational level complies with 12 or 13 years of schooling and a school-leaving qualification, which opens access to higher education institutions [36, 37].

Four parents of children with ASD, recruited from an outpatient department of child and adolescent psychiatry not participating in the study, pretested an initial version of the survey. On the basis of their comments, minor changes to the wordings of questions were made.

### Data analyses

Baseline data were analysed using descriptive statistics. Mean ages with standard deviation (SD) concerning the diagnostic process were determined in the study population by age (0–11, ≥ 12 years), sex (male, female), ASD diagnosis (childhood autism, atypical autism, Asperger syndrome), intellectual functioning (no intellectual disabilities, intellectual disabilities), ADOS-2 comparison score (minimal till low, moderate, high) and highest parental educational level (low/medium, high). Regarding the classification of ASD diagnoses in this study, it should be noted that, unlike the DSM-5, the ICD-10 has not yet incorporated the concept of autism as a “spectrum disorder”, and therefore offers different diagnostic categories for patients with autism.

Multivariable linear regression analyses were in each case performed to evaluate the associations between the above mentioned variables and the age at diagnosis as well as the age at first concerns. A logistic regression assessed the influence of several factors on parental satisfaction with the diagnostic process. Sensitivity analyses were performed using multiple imputation for missing values with 10 imputations. All statistical analyses were performed with SAS, version 9.4 (SAS Institute, Cary, USA).

The study protocol was reviewed and approved by the Commission for Impact Assessment Research and Ethics, Carl von Ossietzky University Oldenburg and by the ethic committees of the participating study sites.

## Results

### Baseline characteristics

637 survey documents were sent to parents/legal guardians of children with ASD, of which 49 documents could not be delivered due to a wrong address. 217 persons returned their questionnaire including a signed written consent (response: 36.9%) and 211 questionnaires could be evaluated. Of these, four questionnaires had to be excluded because of implausible answers, resulting in a final sample of 207 respondents. 39.5% of the non-responders and 34.3% of the responders were parents of children under 12 years. The baseline characteristics are presented in Table 1.

**Table 1:** Baseline characteristics

Characteristics (number of respondents^a^)	n	(%)
Sex (n = 207)		
Male	173	(83.6)
Female	34	(16.4)
Mean age, in years (± SD; range)	12.9	(3.4; 3–18)
Age groups in years (n = 207)		
0–11	71	(34.3)
≥ 12	136	(65.7)
Diagnoses (n = 207)		
Childhood autism (F84.0)	115	(55.6)
Atypical autism (F84.1)	42	(20.3)
Asperger syndrome (F84.5)	50	(24.2)
Intellectual functioning (n = 172)		
IQ ≥ 85	97	(56.4)
IQ < 85	75	(43.6)
ADOS-2 comparison score (n = 188)		
Minimal to low (1–4)	27	(14.4)
Moderate (5–7)	95	(50.5)
High (8–10)	66	(35.1)
Highest parental level of education (n = 206)		
Low/middle	80	(38.8)
High	126	(61.2)
Respondents’ relationship to the child (n = 206)		
Mother	123	(59.7)
Father	18	(8.7)
Both parents	57	(27.7)
Grandparents or other relatives	2	(1.0)
Foster or adoptive parents	6	(2.9)

^a^Due to missing values, figures may differ

### Age at first concerns

On average, parents reported that they first had concerns about their child’s development when their child was 23.4 months old. 39.0% of parents noted developmental atypicalities during the 1st year of life, and one-fourth became concerned during 13 and 24 months (25.4%). Parents of children in the older age group first suspected a difference in the development of their child later than parents of younger children (25.5 vs. 19.4 months) (Table 2). Parents of children with a diagnosis of childhood autism were first concerned at a mean age of 21.3 months whereas parents of children with Asperger syndrome were concerned later (29.8 months). Developmental abnormalities were noted earlier among children with an IQ < 85 compared to children with an IQ ≥ 85 (17.5 vs. 30.2 months).

**Table 2:** Mean age at first concerns and at diagnosis and mean time taken from first concerns to diagnosis stratified by characteristics

Characteristic	Age at first concerns		Age at diagnosis		Time from first concerns to diagnosis	
	N	Mean in months (SD)	N	Mean in months (SD)	N	Mean in months (SD)
Sex						
Male	171	24.0 (18.9)	170	78.3 (37.5)	169	54.8 (36.6)
Female	34	20.8 (14.8)	33	79.6 (43.1)	33	58.5 (44.2)
Age groups in years						
0–11	69	19.4 (15.1)	70	55.7 (21.6)	69	36.5 (24.5)
≥ 12	136	25.5 (19.5)	133	90.5 (39.9)	133	65.2 (39.9)
ASD diagnosis						
Childhood autism (F84.0)	114	21.3 (16.4)	113	68.1 (35.4)	112	47.3 (34.2)
Atypical autism (F84.1)	42	21.7 (15.2)	41	83.9 (42.0)	41	61.9 (43.6)
Asperger syndrome (F84.5)	49	29.8 (23.2)	49	98.1 (33.7)	49	68.3 (36.7)
Intellectual functioning						
IQ ≥ 85	95	30.2 (21.2)	94	89.3 (36.6)	93	59.9 (37.1)
IQ < 85	75	17.5 (13.3)	75	73.8 (38.5)	75	56.3 (38.8)
ADOS-2 comparison score						
Minimal to low (1–4)	27	28.1 (18.7)	25	94.4 (42.5)	25	67.4 (38.3)
Moderate (5–7)	93	22.5 (19.5)	94	79.9 (37.3)	93	57.8 (39.4)
High (8–10)	66	22.0 (15.8)	65	70.9 (37.5)	65	48.8 (34.5)
Highest parental level of education						
Low/middle	80	25.4 (18.1)	79	82.1 (41.0)	79	56.9 (41.7)
High	124	22.3 (18.4)	123	76.0 (36.8)	122	54.1 (35.3)
Overall	205	23.4 (18.3)	203	78.5 (38.4)	202	55.4 (37.8)

Table 3 shows the results of the multivariable linear regression. Adjusting for all other covariates, parents of children in the older age group were concerned 8.4 months later than parents of younger children (p = 0.0084) and parents of children with an IQ < 85 noted atypicalities 13.0 months earlier than parents of children with an IQ ≥ 85 (p < 0.0001). Sex, ASD diagnosis, ADOS-2 comparison score and the parental level of education were not significantly associated with age at first concerns in multivariable analyses. Sensitivity analyses did not lead to any significant changes.

**Table 3:** Potential predictors for age at first concerns and age at diagnosis

Characteristic	Age at first concerns in months^a^		Age at diagnosis in months^a^	
	Crude linear regression β (95% CI)	Multivariable linear regression^b^ β (95% CI)	Crude linear regression β (95% CI)	Multivariable linear regression^b^ β (95% CI)
Sex				
Male	Reference	Reference	Reference	Reference
Female	− 3.19 (− 9.90 to 3.51)	− 0.69 (− 8.38 to 7.00)	1.28 (− 13.00 to 15.56)	9.67 (− 4.33 to 23.68)
Age groups in years				
0–11	Reference	Reference	Reference	Reference
≥ 12	6.09 (0.86 to 11.31)	*8.42 * (*2.16 to 14.68*)	*34.83 * (*24.84 to 44.83*)	*38.23 * (*26.92 to 49.52*)
ASD diagnosis				
Childhood autism (F84.0)	Reference	Reference	Reference	Reference
Atypical autism (F84.1)	0.37 (− 5.96 to 6.71)	− 2.05 (− 9.22 to 5.12)	*15.83 * (*2.91 to 28.76*)	*19.66 * (*6.58 to 32.73*)
Asperger syndrome (F84.5)	*8.41 * (*2.42 to 14.41*)	2.52 (− 4.94 to 9.97)	*29.99 * (*17.86 to 42.12*)	*19.35 * (*5.71 to 32.98*)
Intellectual functioning				
IQ ≥ 85	Reference	Reference	Reference	Reference
IQ < 85	− *12.72 * (− *18.18 to* − *7.27*)	− *12.96 * (− *19.31 to* − *6.62*)	− *15.51 * (− *26.80 to* − *4.21*)	− *15.53 * (− *27.13 to* − *3.93*)
ADOS-2 comparison score				
Minimal to low (1–4)	Reference	Reference	Reference	Reference
Moderate (5–7)	− 5.61 (− 13.32 to 2.10)	− 2.40 (− 10.17 to 5.36)	− 14.51 (− 31.19 to 2.17)	− 5.91 (− 20.46 to 8.63)
High (8–10)	− 6.14 (− 14.20 to 1.92)	− 3.27 (− 11.38 to 4.84)	− *23.44 * (− *40.88 to* − *5.99*)	− 11.43 (− 26.61 to 3.76)
Highest parental level of education				
Low/middle	Reference	Reference	Reference	Reference
High	− 3.03 (− 8.14 to 2.08)	− 4.65 (− 10.41 to 1.10)	− 6.09 (− 16.90 to 4.72)	− 9.94 (− 20.50 to 0.62)

Significant values are shown in italics

^a^ Sample size varies depending on missing values

^b^ Adjusted for all other variables shown

### Age at diagnosis

In our sample, children received their ASD diagnosis at a mean age of 78.5 months (6.5 years) and 30.1% received the diagnosis during the first 48 months. Children in the younger group were diagnosed considerably earlier than children older than 11 years at the time of the survey (Table 2). Whereas children with childhood autism received their diagnoses at a mean age of 68.1 months (5.7 years), children with atypical autism and Asperger syndrome were diagnosed significantly later (at age 83.9 and 98.1 months, respectively). Diagnoses for children with an IQ < 85 were made much earlier than for children with an IQ ≥ 85. Children with a high ADOS-2 comparison score tended to be diagnosed earlier than children with a minimal till low score. Differences between the mentioned subgroups were statistically significant in the crude linear regression (Table 3).

Multivariable linear regression showed that children aged > 11 years (at the time of the survey) were diagnosed 38.2 months later than younger children (p < 0.0001). Compared to children with childhood autism, children with atypical autism received their diagnosis 19.7 months later, and those with Asperger syndrome were diagnosed 19.35 months later (p = 0.0032, p = 0.0054). Children with an IQ < 85 were diagnosed 15.5 months earlier than children with an IQ ≥ 85 (p = 0.0087). The remaining hypothesized predictors seen in Table 3 were not significantly associated with age at diagnosis in multivariable analyses, with sensitivity analyses yielding no significant changes.

The average time from first parental concerns and the establishment of a definite ASD diagnosis was 55.4 months (Table 2). Children aged > 11 years at the time of the survey experienced a longer diagnostic delay than children in the younger group. The mean interval from concerns to definite diagnosis was longer for children with atypical autism (61.9 months), or Asperger syndrome (68.3 months), when compared to children with childhood autism (47.3 months).

### Referral trajectories

Because of the first noted abnormalities with their child’s development, the majority of parents first consulted a paediatrician (74.1%). Only a minority of parents reported that they directly sought help at a social-paediatric centre (7.5%), or a specialist for child and adolescent psychiatry (4.0%). The outcome of the first consultation was mixed: 30.1% of parents were referred to another professional, and 27.6% were told that there was no problem with their child (“don’t worry” or “he’ll grow out of it”). In 17.2% of the cases a different diagnosis than ASD was given, and 11.3% were told to return if the problems persisted. The percentage of cases, where the final diagnosis was already made at the first consultation, was 7.4%.

On average, parents visited 3.4 different professionals (SD = 2.4, range 1–20, median: 3.0) until their child received an ASD diagnosis. The diagnosis was most frequently made at a special outpatient clinic for ASD (48.5%), or at an autism treatment centre (19.3%).

### Satisfaction with the diagnostic process

38.5% of respondents were satisfied, 38.0% were dissatisfied and 23.4% were neither satisfied nor dissatisfied with the diagnostic process. Dissatisfaction was more frequent among parents of children with an IQ < 85, compared to those with an IQ ≥ 85. Prevalence of dissatisfaction was highest among parents of children with low ADOS-2 comparison scores, and decreased with increasing scores. Percentage of dissatisfaction increased with the number of professionals seen so get an ASD diagnosis (see Additional file 1).

The Additional file 1 shows the results of the logistic regression. Adjusting for all other covariates, the only factor significantly associated with dissatisfaction with the diagnostic process is the number of professionals seen during the process of obtaining a diagnosis. There were no significant changes in sensitivity analyses.

## Discussion

In this study, children received their ASD diagnosis on average at an age of 6.5 years, with an interval of 4.6 years from first parental concerns to diagnosis. This finding is in line with a previous German study [30], that reported a mean age at ASD diagnosis of 6.3 years. Similar to our results (23.4 months), the Noterdaeme et al. study also found that the majority of parents became concerned of their child’s development during the 2nd year of life. In the retrospective study of Kamp-Becker et al. [14], who analysed a cohort of children with Asperger syndrome in Germany, the mean age at diagnosis was much higher, i.e. 11.6 years. In 47.3% of cases, parents had first concerns regarding their child’s development within the first 2 years of life, and in 43.2% concerns arose between age 3 and 6. These results correspond to the observed differences between the ASD subgroups in this study. While in our study the mean age at diagnosis for children with Asperger syndrome was more than 3 years lower than in the study of Kamp-Becker et al. [14], children with Asperger syndrome were likewise diagnosed considerably later than children with other ASD diagnoses in our study. The lower age at diagnosis for children with Asperger syndrome in our study may also be a result of the more recent recruitment time period, in which both public and professional awareness of ASD, including Asperger syndrome, has increased.

In the multi-country European study of Salomone et al. [29] mean age at diagnosis was 3.5 years overall, and 4.0 years in Germany. The difference to our study, where the mean age at diagnosis was 6.5 years, can be explained by the different study designs: While the Salomone et al. study was a web-based survey, our study was clinic-based, thus having a bias towards more complex cases, which may be associated with higher age at diagnosis. Furthermore, Salomone et al. only recruited younger children (4 to 7 years of age), thus limiting age at diagnosis to a max. of 7 years, and probably also excluding most children with Asperger syndrome, who are often diagnosed significantly later. In contrast, our study had a broader age range, and our sample included a significant portion of patients with Asperger syndrome, resulting in higher age at diagnosis. Nevertheless, both in the Salomone et al. and in the current study, Germany is among those countries where age at diagnosis is highest.

In the review of Daniels and Mandell [13], who explored 42 studies from the period 1990–2012, in only three studies (one from France, one from the Czech Republic, and an older one from the UK) the average age at ASD diagnosis was higher than 6.5 years. In more recent studies, the age at ASD diagnosis was 4.6 years in a report from the UK [38], 3.9 years in a study from Norway [39], 4.4, 5.4 and 7.4 years, respectively, in three surveys from the USA [40, 41], and 6.2 years in a study from the Czech Republic [42].

### Time until diagnosis

While in line with former studies from Germany, the comparably long time until diagnosis found in this study is nevertheless somewhat surprising, as Germany has a high-quality health system with generally good access to child and adolescent mental health services. There is a range of possible explanations for these findings, that encompass parental, professional, and health system factors. One possible reason is given in a study from the UK by Crane et al. [16], where parents usually waited a year from first concerns to initiating contact with a health professional. Waiting times for diagnostic appointments may also contribute to delayed diagnoses: In a Canadian study, the median waiting time for an ASD-specific diagnostic assessment was 7 months [43]. This also applies to Germany—as a result of the increased public awareness of ASD and the resulting requests for diagnostic ASD evaluations [4], the few existing specialised clinics for ASD in Germany are fully booked up, and long waiting lists are common. Unwarranted referrals also take up diagnostic capacities, as an US study by Monteiro et al. [44] demonstrated. In their study, a substantial portion of children without ASD were referred to scarce autism diagnostic resources, potentially delaying diagnosis and subsequent access to services for children who truly had an ASD [45].

The above-mentioned Canadian study [43] offers another reason for delays within the diagnostic process: The use of insufficient diagnostic tools, or of no diagnostic tools at all, may make subsequent consultations of further professionals necessary. The significant proportion of participants in our study with either Asperger syndrome (usually diagnosed much later than other forms of autism), or less pronounced ASD symptoms (more difficult to diagnose), may also have increased the average time to diagnosis. Additionally, all study participants came from specialized outpatient ASD clinics, which usually care for the most complex or difficult to diagnose patients. As this usually takes longer than in uncomplicated cases, this factor is another explanation for the longer time to diagnosis.

On a health system level, the prominent place of primary care physicians in the Germany health system [46] may also have contributed to the delay until diagnosis. In a UK study, nearly 40% of general practitioners had no substantial knowledge on ASD and the respective referral pathways [47]. In Germany, as mentioned above, no established referral pathways for children with suspected ASD exist, which may have contributed to both longer time to diagnosis, and to lower diagnostic quality [2]. The geographical settings of the participating centres may also have had an effect on age at ASD diagnosis [13], as in Germany the respective federal states differ significantly in terms of mental health resources [48].

### Factors associated with earlier diagnosis

In our study, factors associated with an earlier age of diagnosis were childhood autism and learning disability or intellectual disability, respectively. While these factors largely comply with the German study from Noterdaeme et al. [30], as well as with the international literature [13], other well-known influencing factors like parental education were not significantly associated with earlier diagnosis in our study.

### Referral trajectories

Regarding the number of professionals consulted until the final ASD diagnosis (3.4), comparable studies are scarce. Noticeably, the number of consulted professionals is closely related to the peculiarities of each national health system, and especially to its referral patterns. In an older study from the UK, Goin-Kochel et al. [15] reported an average of four to five (range 1–29) contacts to different clinicians on their way towards an ASD diagnosis, which is largely similar to our data. However, the fact that in our study on average it took 4.6 years from first parental concerns to diagnosis while parents consulted “only” 3.4 professionals during this period, is difficult to explain.

Nevertheless, the broad range of contacts with professionals in our study (up to 20) indicates a significant number of parents that have followed intertwined diagnostic pathways. In about half of patients in our sample, the definite diagnosis was made at a specialised ASD outpatient clinic. While this might reflect a recruitment bias, this proportion fits well with the figures of the web-based study of Goin-Kochel et al. [15], who reported the percentage of diagnoses at a specialist doctor at 46.8%.

### Satisfaction with the diagnostic process

The finding of nearly 40% dissatisfied parents is in line with the data from other surveys: In a study from the UK by Goin-Kochel et al. [15], 40.1% of parents were not satisfied with the diagnostic process. Similarly, Jones et al. [49], who studied a sample of adults with ASD from the UK, found 40% of respondents to be very dissatisfied or quite dissatisfied. In a more recent study, Crane et al. [16] even found more than half of patients dissatisfied with the diagnostic process. A French survey by Chamak et al. [50] that evaluated parents’ satisfaction with the way the ASD diagnosis was announced, yielded dissatisfaction rates of 63% (children), and 93% (adults), respectively.

Regarding influencing factors, in our study only the number of professionals seen was associated with dissatisfaction with the diagnostic process, while age, level of education, or diagnostic subgroup were not. This is in contrast to other studies, who found a broader list of associated factors, including time to diagnosis, maternal education, manner of the diagnosing professional and family income [15, 16, 51], and might be explained by the smaller sample size of our study.

### Strengths and limitations

A strength of this study is the sample of children and adolescent with ASD diagnoses, which have been established using high-quality diagnostic standards [22]. An important limitation of this study is the low response rate, which limits generalisation of results. The relatively small sample size might have led to lower statistical power and large confidence intervals in multivariable analyses, and the wide range of patients’ age in our sample caused large standard deviations for age at diagnosis and time to diagnosis. Due to the recruitment through specialized ASD outpatient clinics, our sample may be biased towards more complicated cases, which in turn might be associated with longer times to the definite ASD diagnosis. Because of the significant period between ASD diagnosis and this survey, there is also a risk for recall bias in parents’ responses. For the sake of brevity, our questionnaire did not ask for e.g. ethnicity, comorbidity or family structure, which in some studies were factors associated with time to diagnosis [13, 52]. Moreover, our study focused on the diagnostic process, thus missing out on the equally important post-diagnosis phase, e.g. provision of information on ASD-specific services [16], or time to receiving services [53]. The cross-sectional study design precluded an analysis of potential period effects, which have been reported in other studies [13, 38, 50], and may have affected the time to diagnosis in our study.

## Conclusions

Concluding, our study shows that in the studied sample of children with ASD in Germany the time to diagnosis was higher than in the majority of international studies. This is regrettable, as a timely diagnosis is important in children with ASD, in order to enable them and their families to make use of social support and health services, and to improve their vocational outcome [54].

The results flag the need for better screening algorithms in primary care in Germany, especially for paediatricians, who were the first point of contact in three quarters of all cases in our study. Additionally, there is a need for clear referral criteria for children with suspected ASD, that allow a quicker and easier diagnostic process [55], in order to decrease time to diagnosis and increase parental satisfaction. The newly published German guidelines on diagnosis in suspected cases of ASD [26] will hopefully contribute to this aim. They recommend a two-step approach with first a diagnostic screening by healthcare professionals with experience in developmental disorders and second, in cases with substantiated suspicion, a referral to a specialised ASD outpatient clinic.

## Additional file

**Additional file 1.** Prevalence of dissatisfaction of parents with the diagnostic process and factors associated with dissatisfaction.

## Abbreviations

ADI-R: Autism Diagnostic Interview-Revised
ADOS: Autism Diagnostic Observation Schedule
ASD: autism spectrum disorder
CI: confidence interval
CSRI: Client Service Receipt Inventory
DSM-5: Diagnostic and Statistical Manual of Mental Disorders, Fifth Edition
ICD-10: International Statistical Classification of Diseases and Related Health Problems, 10th Revision
ISCED: International Standard Classification of Education
SAS: Statistical Analysis Software
SD: standard deviation
UK: United Kingdom
USA: United States of America
