# Supplementary material for: Pathways to a diagnosis of autism spectrum disorder in Germany: a survey of parents
Source: Child Adolesc Psychiatry Ment Health. 2019 Mar 21;13:16. doi: 10.1186/s13034-019-0276-1 (PMC6429704; doi:10.1186/s13034-019-0276-1)
Supplement: Supplementary file 1 — Additional file 1. Prevalence of dissatisfaction of parents with the diagnostic process and factors associated with dissatisfaction. [file 13034_2019_276_MOESM1_ESM.docx]

Additional file 1 Prevalence of dissatisfaction of parents with the diagnostic process and factors associated with dissatisfaction

| Characteristic | % not satisfied ^a^ | Crude logistic regression  Odds Ratio (95% CI) | Multivariable logistic regression   Odds Ratio ^b^ (95% CI) |
| --- | --- | --- | --- |
| Sex |  |  |  |
| Male | 37.4 | Reference | Reference |
| Female | 41.2 | 1.17 (0.55-2.48) | 0.78 (0.25-2.45) |
| Age groups in years |  |  |  |
| 0-11 | 37.1 | Reference | Reference |
| ≥ 12 | 38.5 | 1.06 (0.58-1.92) | 0.82 (0.27-2.47) |
| ASD diagnosis |  |  |  |
| Childhood autism (F84.0) | 35.1 | Reference | Reference |
| Atypical autism (F84.1) | 35.7 | 1.03 (0.49-2.15) | 0.89 (0.28-2.78) |
| Asperger syndrome (F84.5) | 46.9 | 1.64 (0.83-3.23) | 0.82 (0.25-2.68) |
| Intellectual functioning |  |  |  |
| IQ≥85 | 46.3 | **2.54 (1.32-4.91)** | 2.01 (0.73-5.53) |
| IQ<85 | 25.3 | Reference | Reference |
| ADOS-2 comparison score |  |  |  |
| minimal to low (1-4) | 50.0 | 2.47 (0.97-6.30) | 3.13 (0.90-10.94) |
| moderate (5-7) | 40.4 | 1.68 (0.86-3.29) | 1.97 (0.76-5.10) |
| high (8-10) | 28.8 | Reference | Reference |
| Highest parental level of education |  |  |  |
| low/middle | 36.3 | Reference | Reference |
| high | 38.7 | 1.11 (0.62-1.99) | 1.32 (0.52-3.35) |
| Age at diagnosis |  |  |  |
| ≤ 48 months | 29.5 | Reference | Reference |
| 49-72 months | 37.2 | 1.42 (0.62-3.24) | 1.10 (0.31-3.84) |
| 73-96 months | 43.9 | 1.87 (0.82-4.27) | 0.96 (0.24-3.84) |
| > 96 months | 43.1 | 1.81 (0.85-3.86) | 1.56(0.37-6.53) |
| Number of professionals seen to get a diagnosis |  |  |  |
| 1-2 | 21.2 | Reference | Reference |
| 3 | 39.3 | **2.40 (1.08-5.34)** | 1.62 (0.61-4.34) |
| ≥4 | 66.0 | **7.21 (3.14-16.55)** | **8.31 (2.77-24.87)** |
|  |  |  |  |

^a^ “very dissatisfied” and “quite dissatisfied”

^b^ adjusted for all other variables shown
